# First report of *Vandellia* sp. parasiting the Raspy river stingray *Potamotrygon scobina* in the Amazon basin

**DOI:** 10.1590/S1984-29612025011

**Published:** 2025-04-04

**Authors:** Paulo Arthur Abreu Trindade, Felipe Antônio Silva, Marcos Sidney Brito Oliveira, Carson Allen Jeffres, Marcelo Ândrade, Tommaso Giarrizzo

**Affiliations:** 1 Programa de Pós-graduação em Ecologia Aquática e Pesca – PPGEAP, Núcleo de Ecologia Aquática e Pesca – NEAP, Universidade Federal do Pará – UFPA, Belém, PA, Brasil; 2 Instituto de Engenharia de Pesca, Universidade Federal Rural da Amazônia – UFRA, Belém, PA, Brasil; 3 Programa de Pós-graduação em Biodiversidade Tropical – PPGBIO, Universidade Federal do Amapá – UNIFAP, Macapá, AP, Brasil; 4 Center for Watershed Sciences, University of California, Davis, CA, USA; 5 Centro de Ciências de Pinheiro, Universidade Federal do Maranhão – UFMA, Pinheiro, MA, Brasil; 6 Instituto de Ciências do Mar – LABOMAR, Universidade Federal do Ceará – UFC, Fortaleza, CE, Brasil

**Keywords:** Vampire fish, candiru, ectoparasites, freshwater stingray, Xingu River, Peixe vampiro, candirú, ectoparasitas, arraia de água doce, rio Xingu

## Abstract

This study reports the first record of candiru, *Vandellia* sp. parasitizing the freshwater stingray *Potamotrygon scobina* in the Amazon basin, Brazil. In April 2018, a specimen of *Potamotrygon scobina* was collected by bottom long-term using fish as bait during the monitoring program in the Xingu River. During a routine inspection, a *Vandellia* sp. was observed in the branchial slit of the *Potamotrygon scobina* specimen. The trichomycterid was collected and preserved in 10% formaldehyde followed by preservation in 70% ethanol. This study is the first to record this parasite associated on a stingray in Brazil.

## Resumo

Este estudo relata o primeiro registro de candirú *Vandellia* sp. parasitando a arraia de água doce *Potamotrygon scobina* na bacia amazônica, Brasil. Em abril de 2018, um espécime de *Potamotrygon scobina* foi capturado por meio de espinhel de fundo usando peixe como isca durante o programa de monitoramento no Rio Xingu. Durante uma inspeção de rotina, uma *Vandellia* sp. foi observada na fenda branquial do espécime de *Potamotrygon scobina.* O trichomycterídeo foi coletado e preservado em 10%, seguido de preservação em etanol 70%. Este estudo é o primeiro a registrar este parasita associado a uma arraia no Brasil.

Candirus belong to the family Trichomycteridae, which comprises eight subfamilies and approximately 45 valid genera, with 357 species (WoRMS, 2025). *Vandelliinae* comprises four genera (*Paracanthopoma*, *Paravandellia*, *Plectrochilus*, and *Vandellia*), in which *Vandellia* comprises only three valid species (*Vandellia cirrhosa*, *Vandellia beccarii*, and *Vandellia sanguinea*) (Dagosta & De Pinna, 2021; WoRMS, 2025). They are obligate hematophagous animals, reaching sizes between 2 and 20 centimeters in total length, expelling large amounts of mucus, which facilitates their entry into small cavities such as gill openings, characteristics that are related to their feeding behavior (De Pinna, 2013).

Candirus are known to parasitize mainly fish, in addition, there are reports of *Ochmacanthus* sp., a Candiru member of the subfamily Stegophilinae, parasitizing the tegument of the freshwater dolphin *Inia geoffrensis* (Araújo‐Wang et al., 2019). Cadirus are also known to cause serious problems to humans due to their ability to enter the urethra (van Ophoven & de Kernion, 2000), however, humans are not natural hosts for these parasites (Bauer, 2013). Candirus are usually found parasitizing the head area, specifically attached to the gill due to the increased vascularization in this area (Spotte et al., 2001; Araújo‐Wang et al., 2019). *Vandellia* are incapable of actively sucking blood, so they make an incision with their opercular spines in the blood vessels of gills and feed as the host fish is actively bleeding (Zuanon & Sazima, 2004a).

**Figure 1:**
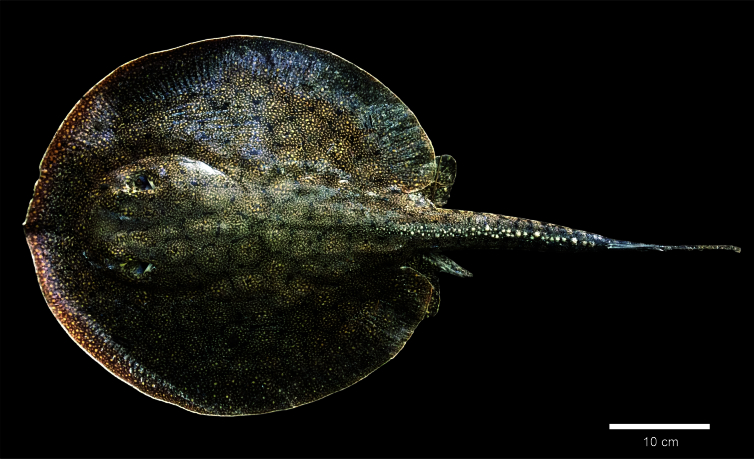
Specimen of *Potamotrygon scobina* collected in Xingu River, Pará State, Brazil. Photo by Paulo Trindade.

South American freshwater stingrays are included in a single family (Potamotrygonidae), represent an important part of the Neotropical ichthyofauna and belong to the only group of elasmobranchs completely restricted to freshwater habitats (Fontenelle et al., 2021). Freshwater stingray species have been regularly captured for ornamental purposes for decades and can be used as a subsistence food source (Lucifora et al., 2022). Potamotrygonidae is a family of 40 valid species of freshwater stingrays found only in the South American rivers that drain to the Atlantic Ocean and Caribbean Sea (Loboda et al., 2021; Fricke & Fong, 2024). Many species are endemic to a single basin, however, species such as *Paratrygon aiereba* (Müller & Henle 1841), *Potamotrygon motoro* (Müller & Henle 1841), *Potamotrygon orbignyi* (Castelnau 1855) and *Potamotrygon scobina* Garman 1913 have broad distribution and they are found in almost all tributaries of the Amazon River (Loboda & Carvalho, 2013; Shibuya et al., 2016). Here we present the first report of the candiru *Vandellia* sp. parasitizing the freshwater stingray *Potamotrygon scobina* (Figure 1).

**Figure 2:**
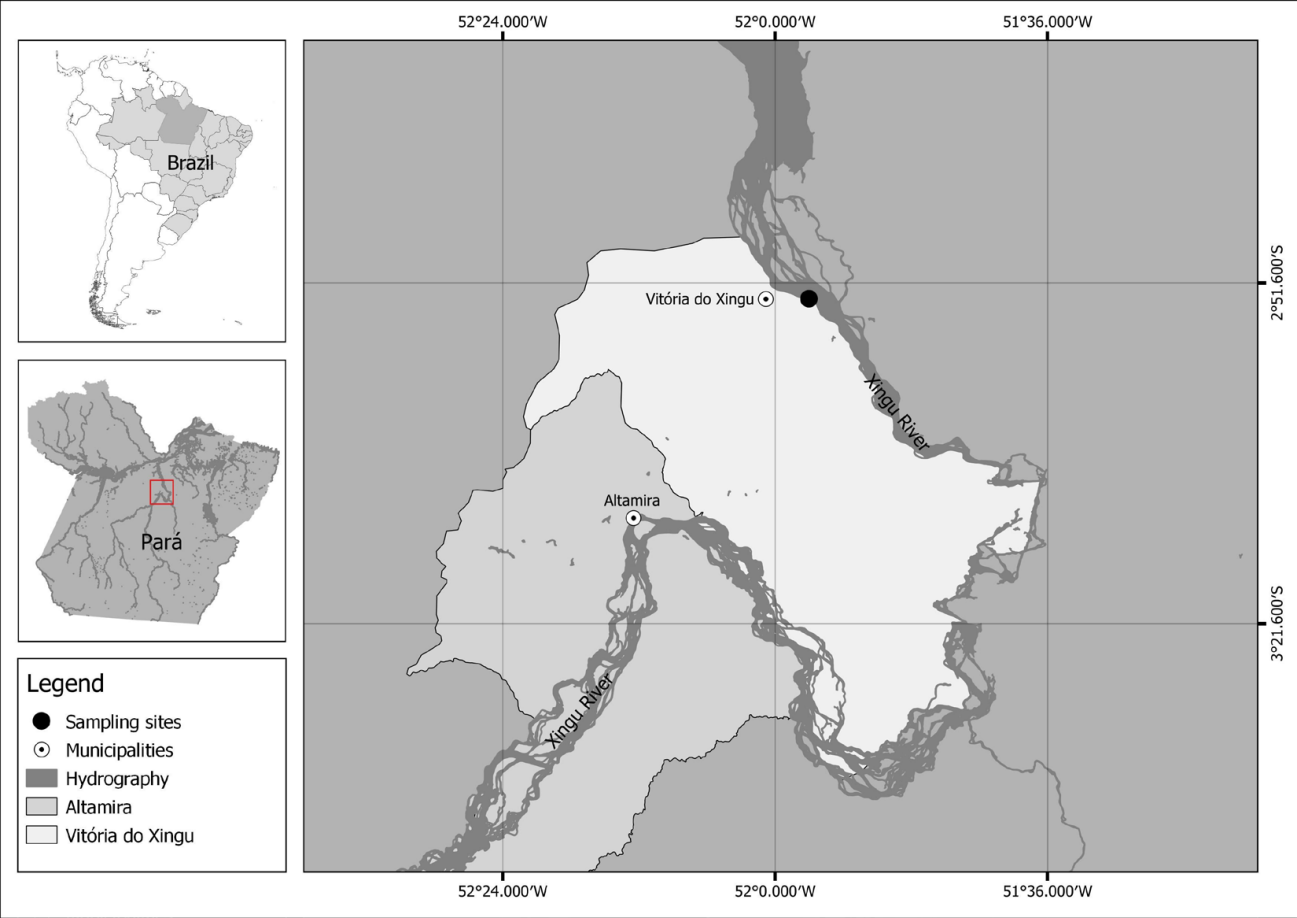
Sampling site of the specimen of *Potamotrygon scobina* parasitised by *Vandellia* sp., in Xingu River, Pará State, Brazil (02°53’37.18” S; 051°56’15.87” W).

The observation was made in April 2018 in the Xingu River, at municipality Vitória do Xingu (02°52’59.4” S; 051°57’01.2” W), Pará State, Brazil (Figure 2). The Xingu River is a clearwater tributary in the lower Amazon River Basin, flows for approximately 2,300 km before entering the Amazon River and housing other species of *Potamotrygon* such as *Potamotrygon leopoldi* Castex & Castello 1970, the endemic one to Xingu River Basin, besides that *Potamotrygon orbignyi*, *Potamotrygon motoro*, and the *Paratrygon aiereba*. Although the Xingu River is known as place where *Potamotrygon scobina* occurs, the parasitism record and specimen identification were made in the field with the posterior live specimen released into the river, and due to evidence of hybridization between *Potamotrygon scobina* and its congeners (Sanches et al., 2021), we decided to maintain the identification as *Potamotrygon scobina*, since the occurrence of the species was described and also related to the Xingu basin by Sousa et al. (in press).

The specimens were collected under permit number 02001.011114/2020-52 issued by the Instituto Brasileiro do Meio Ambiente e dos Recursos Naturais Renováveis (IBAMA) as part of an ichthyofauna monitoring program for the construction of the Belo Monte Hydroelectric Plant. In April 2018, a specimen of *Potamotrygon scobina* was collected by a bottom long-line using fish as bait. A standard protocol was used to obtain total length (TL), standard length (SL), disc width (DW) in centimeters (cm), total weight (W) in grams (g) and sex was determined. The candiru was removed from the gill slit on the ventral surface of the ray and preserved in 10% formaldehyde followed by preservation in 70% ethanol. Upon returning to the laboratory, the candiru was measured (SL), identified and cataloged in the Laboratório de Ictiologia do Grupo de Ecologia Aquática (GEA 7331) of the Universidade Federal do Pará (UFPA), Belém, Pará state, Brazil.

**Figure 3:**
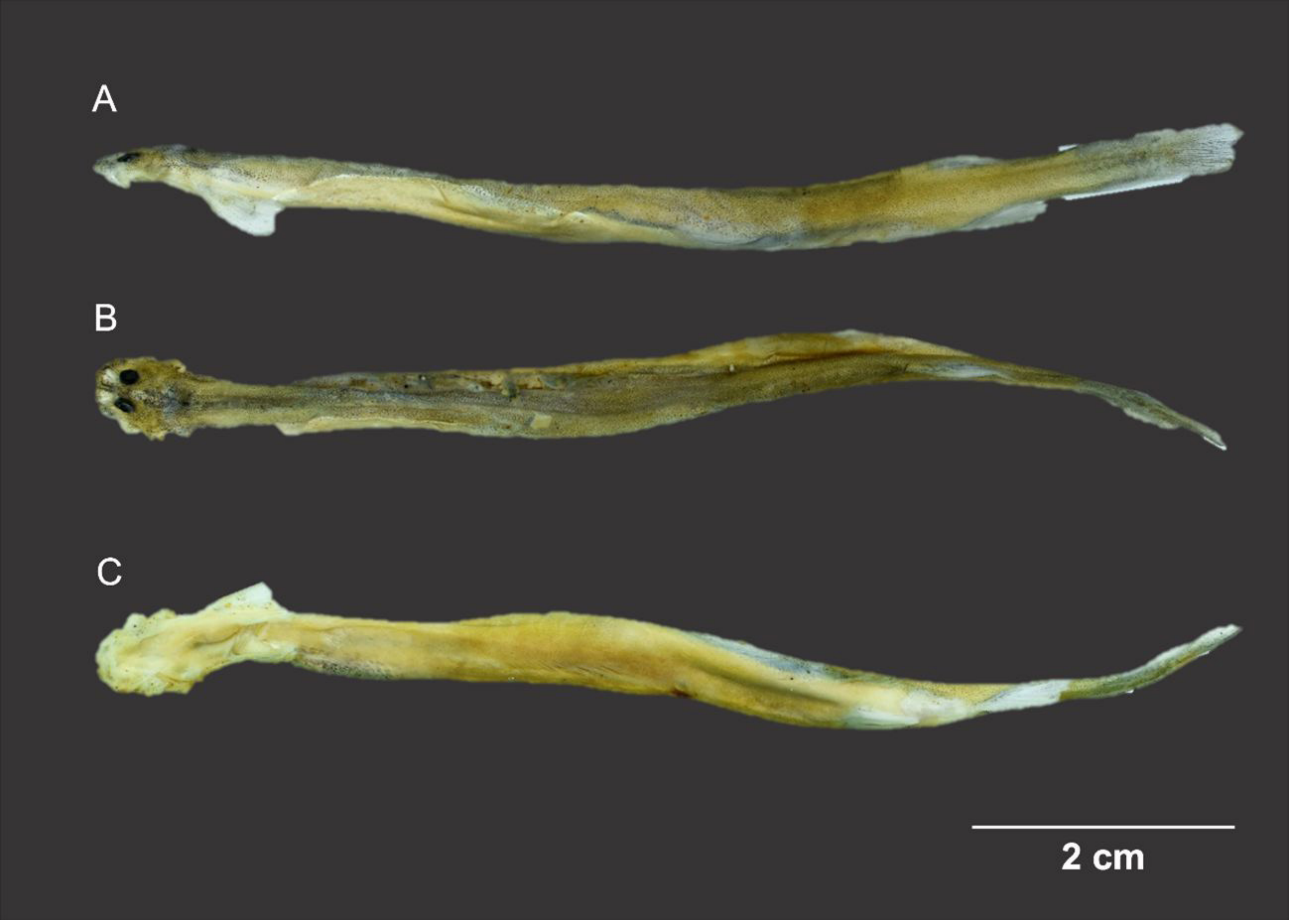
Lateral (A), dorsal (B) and ventral (C) views of the Candiru *Vandellia* sp. collected from the stingray host. Photo by Marcelo Ândrade.

The stingray (host) was an adult male of 80 cm TL, 55 cm SL, 50.2 cm DW and 6.600 g. The candiru was identified as a *Vandellia* sp. (Figure 3) with 8.75 cm SL was found attached in a branchial slit of the Stingray.

The candiru was identified as a hematophagous candiru of the genus *Vandellia* Valenciennes 1846. As described by Ohara et al. (2017) they have a very elongated body shape, small mouth lacking a suction cup shape like other members of the Trichomycteridae family; teeth arranged in irregular rows and concentrated in the middle of the upper jaw.

In South America, there are records of 75 different parasites that parasitize one or more species of freshwater stingrays (Gama, 2016). The parasites belong to the groups Cestoda, Monogenea, Digenea, Sporozoa, Nematoda, Acanthocephala, Ciliophora, Crustacea, Pentastomida and Pisces (Gama, 2016). This study has found that the family Potamotrygonidae is frequently parasitized by several groups of parasites on different locations of the body. Location of parasitism depends on the feeding mechanism and life cycle of the parasite, and they can be found both internal and external on the host.

For Potamotrygonidae, there are records of *Vandellia beccarii* (Di Caporiacco 1935) parasitizing *Potamotrygon orbignyi* and *Paracanthopoma* (Giltay 1935) in *Potamotrygon* sp. in the Orinoco River, near the border between Colombia and Venezuela (Lasso et al., 2015). While for the Xingu River, the only records of parasites in stingrays were the Cestoda *Rhinebothroides freitasi* (Rêgo, 1979) Brooks, Mayes & Thorson, 1981 in *Potamotrygon leopoldi* (Marques & Brooks, 2003); the Monogenea and *Potamotrygonocotyle aramasae* Domingues, Pancera & Marques, 2007 from *Paratrygon aiereba* (Müller & Henle, 1841). However, none of these studies recorded the occurrence of candirus parasitizing freshwater stingrays in Brazil.

Because they are small and almost transparent, candirus attached to hosts are provided a level of protection from predators while they are attached to the host (De Pinna, 2013). In addition to parasitizing and protecting itself from predation, host migrations can greatly extend the potential range of the relatively small candirus (Lubich et al., 2021). *Paracanthopoma* sp. have been observed attached to the body of the large migratory fish, the Gilded catfish *Zungaro zungaro* (Humboldt 1821). Due to the large migratory distances of *Z. zungaro*, it is hypothesized that this behavior could facilitate the dispersal of candiru across the Amazon basin (Zuanon & Sazima, 2005). Unlike the observations of *Vandellia* sp. on long-distance migratory hosts, the observed parasitism of *Potamotrygon scobina*, is not considered a mechanism of wide dispersal like *Z. zungaro* (Zuanon & Sazima, 2005). Furthermore, it is known that Potamotrygonidae do not perform large migrations, only bathymetric movements (Garrone & Uieda, 2012). Despite the lack of potential for long-range facilitated migration, candirus could still benefit from the host’s short-range movements, saving energy while moving unnoticed to predators (Lasso et al., 2015). In general, stingrays are benthic organisms that spend much time resting or buried on the river bottom, preferentially in sandy or muddy substrate where prey capture occurs via rapid uplift of the pectoral fins, exposing prey beneath the ray’s body (Lasso et al., 2015; Kolmann et al., 2016). Vandelliinae are often found in similar substrate as stingrays so it is not surprising that the two species could occasionally interact (Zuanon & Sazima, 2004b). We conclude that this is the first record of *Vandellia* sp. parasitizing freshwater stingrays in Brazil. Furthermore, this study increases knowledge of the number of hosts that candirus use as hosts in natural systems.
